# Using self-monitoring to detect and manage raised blood pressure and pre-eclampsia during pregnancy: the BUMP research programme and its impact

**DOI:** 10.1038/s41440-023-01474-w

**Published:** 2023-12-07

**Authors:** Katherine L. Tucker, Lisa Hinton, Marcus Green, Lucy C. Chappell, Richard J. McManus

**Affiliations:** 1 Nuffield Department of Primary Care Health Sciences, University of Oxford, Oxford, UK; 2 The Healthcare Improvement Studies (THIS) Institute, University of Cambridge, Cambridge, UK; 3 Action on Pre-Eclampsia (APEC), Charity, Worcestershire, UK; 4 Department of Women and Children’s Health, School of Life Course Sciences, King’s College London, London, UK; 5 Present Address: Nuffield Department of Primary Care Health Sciences, University of Oxford, Oxford, UK

**Keywords:** Self-monitoring, Hypertension, Pregnancy, Blood pressure, Pre-eclampsia

## Abstract

Raised blood pressure affects around ten percent of pregnancies worldwide, causing maternal and perinatal morbidity and mortality. Self-monitoring of blood pressure during higher-risk or hypertensive pregnancy has been shown to be feasible, acceptable, safe, and no more expensive than usual care alone. Additionally, self-testing for proteinuria has been shown to be just as accurate as healthcare professional testing, creating the potential for monitoring of multiple indicators through pregnancy. The work suggests however, that an organisational shift is needed to properly use and see benefits from self-monitored readings. This paper describes the findings from a large programme of work examining the use of self-monitoring in pregnancy, summarising the findings in the context of the wider literature and current clinical context.

**Figure:**
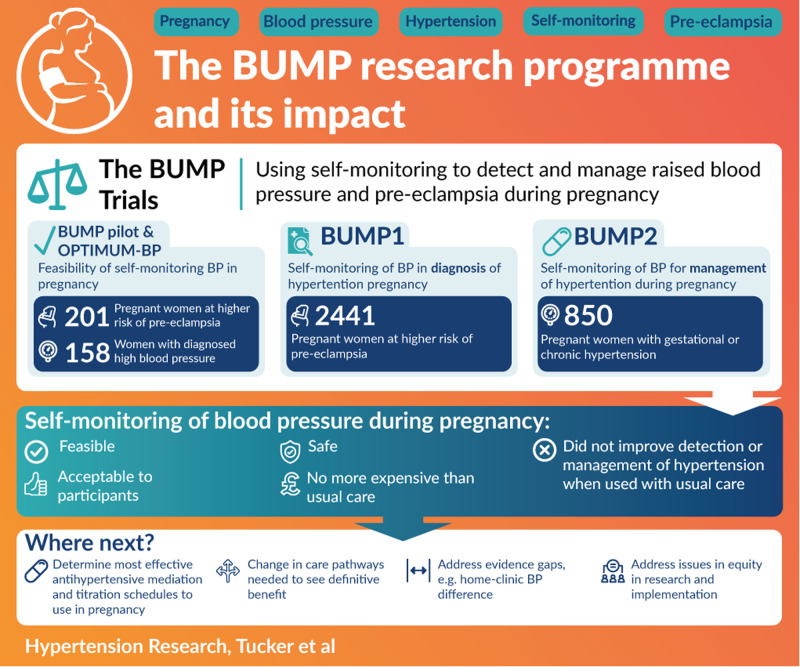
The BUMP Research Programme developed and tested self-monitoring and self-testing interventions for pregnancy. The work showed that self-monitoring during pregnancy was feasible, acceptable, safe, and no more expensive, but did not improve the detection or control of hypertension.

The BUMP Research Programme developed and tested self-monitoring and self-testing interventions for pregnancy. The work showed that self-monitoring during pregnancy was feasible, acceptable, safe, and no more expensive, but did not improve the detection or control of hypertension.

## Abstract (web summary)

The BUMP Research Programme developed and tested self-monitoring and self-testing interventions for pregnancy. The work showed that self-monitoring during pregnancy was feasible, acceptable, safe, and no more expensive, but did not improve the detection or control of hypertension.

## Introduction

Raised blood pressure (BP) affects ~10% of pregnancies, and is associated with an increased risk of developing pre-eclampsia, and in the long-term an increased risk of chronic hypertension and cardiovascular disease [1, 2]. The early detection and subsequent management of pregnancy hypertension is important, in order to optimise pregnancy outcomes for the woman and infant. Unlike BP monitoring and management outside of pregnancy, BP can change rapidly during pregnancy and changes can be difficult to predict [1].

Self-monitoring of blood pressure (SMBP), where an individual measures their own BP outside of the clinical setting, is effective at detecting and lowering blood pressure in adults with hypertension, and now commonplace outside of pregnancy [3–5]. In pregnancy and the postnatal period, SMBP has the potential to improve the detection and management of raised BP whilst empowering women and potentially reducing clinic visits [6].

Interest in the opportunities for SMBP in pregnancy and the postnatal period is increasing. In the last five years, several studies have reported research around the use of SMBP during pregnancy, from settings around the world including Europe, Asia and North America. Early work from small observational studies suggested the potential for reduced morbidity and resource use, as well as acceptability and feasibility, yet evidence from large-scale randomised trials for the effectiveness of SMBP in pregnancy has been lacking until recently [7–9]. This article reviews the findings from the Blood Pressure Monitoring in Pregnancy (BUMP) programme of work, undertaken in the United Kingdom (UK), in this rapidly developing area [10–13].

### Prevalence of self-monitoring of blood pressure

A large UK survey in 2019 of pregnant individuals found that overall, around one in five were currently self-monitoring their BP; of those who self-identified as hypertensive, half reported SMBP. However, many of those self-monitoring did not share their readings with their antenatal care team and were not necessarily using pregnancy-validated BP monitors [12]. These findings are in line with a Canadian-based study, which found more than 60% of pregnant women diagnosed with hypertension were already undertaking SMBP [14]. This evidence suggests SMBP has become relatively widespread, and so health care professionals (HCPs) should enquire about SMBP proactively and consider providing information on BP monitoring.

### Self-monitoring thresholds in pregnancy are not clear

A systematic review and individual patient data analysis aimed to explore BP thresholds in pregnancy. The search found over 20 studies and analysed patient data from eight studies (*n* = 758), revealing a mean home-clinic difference of just ≤1.2 mmHg systolic BP throughout pregnancy. However, there was significant heterogeneity between studies (*I*^2^ > 80%); only one study was randomised, and only two of the studies used a monitor validated for use in pregnancy. Although the overall population difference was small (around 2 mmHg systolic), levels of ‘white coat hypertension’ were high, particularly towards the end of pregnancy, when around half of those with clinic hypertension had normal range home readings. The home-office difference was much greater in those with hypertension (predominantly over 10 mmHg systolic through pregnancy) [15]. Data from the OPTIMUM pilot trial and BUMP trials show similar findings [16, 17]. More recent systematic reviews (not including the BUMP trial data) report similar findings with significant clinical heterogeneity [9].

### Trials of self-monitoring of blood pressure

Until recently, few data were available regarding SMBP as an intervention in pregnancy; a systematic review found just one previous randomised controlled trial of self-monitoring in an antenatal setting with normotensive women [18]. This included 80 low-risk pregnancy women who were randomised to undertake either weekly self-monitoring with reduced routine antenatal clinics or usual care (but did not evaluate the impact of self-monitoring on the timing of new diagnoses of pregnancy hypertension). We found one additional trial in which a US group randomised 300 low-risk pregnant women to remote monitoring with reduced clinic visits or usual care. The women randomised to remote care had reduced obstetric input but more nurse/midwife time was needed for the remote care. Other maternal and perinatal outcomes were similar between the groups. The BUMP pilot study and OPTIMUM-BP pilot trial established feasibility in the UK health service of SMBP in higher risk and hypertensive pregnancies and supported the development of the BUMP trials, which aimed to assess the place of self-monitoring in the diagnosis (BUMP1) and management (BUMP2) of hypertension in pregnancy [10, 11].

The BUMP trials were two fully powered randomised controlled trials of SMBP in pregnancy that recruited more than 3000 women at higher risk of pre-eclampsia, or with pregnancy hypertension. Participating women were randomly allocated to either usual care or usual care plus SMBP.

The BUMP1 trial recruited 2441 pregnant women at higher risk of pre-eclampsia around 20 weeks’ gestation with the aim of detecting raised BP earlier than standard clinic visits. The trial found that SMBP during higher-risk pregnancy appeared to be safe, but did not significantly improve earlier clinic-based detection of hypertension when used alongside usual care. Despite this, there was a signal of the potential of SMBP: self-monitoring did provide potential prior notice of hypertension in many women, with 61% (109/179) of those with hypertension reporting raised SMBP around one month prior to clinic diagnosis (median 29 days earlier). This suggests that SMBP in pregnancy could have been used to detect hypertension earlier, but the information was not necessarily acted upon by clinicians (or possibly by women). Further work is required to assess the place of self-monitoring of BP in this higher-risk population, for instance in remote consultations or alongside self-management, and to understand how to better engage HCPs in its use [11].

The BUMP2 trial recruited 850 women with gestational or chronic hypertension, with the aim of improving BP control (as had been found outside of pregnancy) [4]. The intervention of SMBP did not result in improved BP control as assessed by clinic systolic BP but was acceptable, with no difference in adverse maternal or infant outcomes, and was equivalent in cost to usual care alone [19]. As with using monitoring for earlier diagnosis, further co-interventions may be required in order to achieve improvements in BP control and other pregnancy outcomes [10].

For the BUMP2 trial, many women’s average blood pressure was above the NICE target of 135/85mmHgh based on their clinic readings, suggesting additional titration of antihypertensive medication might have been needed [10]. Additionally, a higher proportion than expected in the usual care arm (68%) reported prior SMBP, but these data were not available during the trial itself. These considerations, in combination with the survey results, suggest that SMBP is already part of women’s health behaviours but has not yet been fully or formally incorporated into care pathways [12].

### Understanding the trial findings

A process evaluation of the trials showed that the majority of women and healthcare professionals involved in the trial found that SMBP enhanced their experiences of the clinical encounter and the HCP-woman relationship [20, 21]. However, the pursuit of normal readings, selective or delayed reporting of raised readings, and variable engagement by HCPs with home readings, could have influenced the impact of the BUMP intervention. Interviews and observations suggest that SMBP led to both earlier detection of rising BP, but also was used as justification for not starting intensifying antihypertensive medication when home readings were lower [20, 21]. Finally, SMBP by women in the usual care arm may have reduced between-groups differences, limiting the evidence for the intervention’s effectiveness.

### What helps (or hinders) SMBP in practice?

Qualitative work from linked studies showed that women were highly motivated and empowered by SMBP, reporting greater control, knowledge and reassurance. Interviews and ethnographic observations showed that good communication and effective partnerships between women and clinicians underpinned the use of SMBP, and may in turn have supported shared decision-making [22].

However, uncertainty remains around home-clinic differences and how this information could be translated into clinical decisions, with the qualitative work in the BUMP trials revealing that some clinicians had higher confidence in clinical readings (rather than home readings) when it came to taking action [15, 20].

### Proteinuria self-testing in pregnancy

Linked to the BUMP programme, the Proteinuria Detection In Pregnancy (UDIP) diagnostic accuracy study compared the performance of proteinuria testing by women, midwives and automated colorimetric readers. It showed that pregnant women could detect dipstick proteinuria with similar accuracy to healthcare professionals, suggesting urine self-testing alongside BP self-monitoring would be feasible. Furthermore, the study also showed that automated colorimetric testing was not significantly different to performance by women or healthcare professionals. Although the accuracy of urine dipstick testing is not as high as desired in a diagnostic test, testing in the context of antenatal care, which includes repeated testing, is likely to be beneficial [13]. Furthermore, using protein testing in combination with home BP monitoring could allow better reassurance to both healthcare professionals and women that the important indicators of evolving pre-eclampsia are being monitored.

### The cost-effectiveness of SMBP

Cost-effectiveness analyses of the BUMP 1&2 trials showed that SMBP had no additional cost over and above usual care [19]. The use of SMBP may be better value for money when used for women with pregnancy hypertension than for women at risk of hypertension, due to a greater risk of developing complications both during and following pregnancy in these women. These findings are in keeping with previous smaller cost-effectiveness studies in the Netherlands and the UK [23, 24]. A key issue is provision of validated monitors and in the future they might also be available for post-partum monitoring and beyond as this is often warranted [25–27]. During the COVID-19 pandemic, the National Health Service in England provided some BP monitors for those with hypertensive pregnancy (see below) and some hospitals may have a small number of monitors for loan [28]. However, for many in the UK, the only option for a pregnant woman is to purchase a monitor, which may result in exclusion of some groups.

### Acceptability of SMBP in the BUMP trials

The SMBP studies described above had good uptake, and linked qualitative work has shown that SMBP is acceptable to higher risk and hypertensive pregnant women and their antenatal care teams [7]. A recent survey suggests that most UK obstetricians (96%) now consider SMBP to be part of current antenatal care [29]. While SMBP during pregnancy has been shown to be acceptable, safe, and no more expensive than usual care, the challenge now is to understand how it can be most effectively integrated into existing care pathways and used to make improvements to outcomes.

### Could self-monitoring be used to guide tighter BP control?

The CHIPS international study of targeting diastolic BP as the intervention showed that tighter control of hypertension was associated with fewer severe hypertension episodes and is safe [30]. More recently, the CHAP study has shown that antihypertensive treatment of mild to moderate chronic hypertension improves pregnancy outcomes, without increased detection of babies small for gestational age [31]. There is a clear rationale for managing women’s blood pressure with tight control, targeting a diastolic BP of 85 mmHg. SMBP could support this change in practice towards earlier and more active management, but would require further work in ensuring that women and healthcare professionals understand and take appropriate action on self-monitored BP readings.

### Lessons from rapid implementation of SMBP during the COVID-19 pandemic

The COVID-19 pandemic resulted in an urgent shift from clinic-based maternity care to greater remote monitoring, necessitating the rapid implementation of SMBP. In the UK, guidelines were quickly produced, recommending that SMBP was prioritised for women with hypertension or pre-eclampsia, those with risk factors, or those required to self-isolate [32]. Implementation was further supported by the provision of BP monitors by the National Health Service in England, validated for use in pregnancy. During this time, SMBP was predominantly used to provide additional BP monitoring for hypertensive or high-risk pregnant women, rather than replacing face-to-face visits for normal-risk women. Maternity units and women were positive about monitoring to reduce clinic visits and to give women more control and insight into their own BP. However, there were implementation challenges particularly around embedding SMBP into existing care pathways, interpreting readings and managing the provision of monitors [28]. This revealed a need for further research into appropriate care pathways and information and guidance around management of white coat or masked (high home BP readings, normal clinic readings) hypertension.

These implementation studies are part of a wider emerging evidence base and health system interest in virtual care [33]. The rapid reconfiguration of antenatal services in response to the pandemic has highlighted a significant shift to remote provision, of which SMBP was an integral component [34–36]. A large UK study undertaken during the COVID-19 pandemic, highlighted the potential accessibility and efficiency advantages of remote antenatal care reported by women including saving time, stress, travel expenses and reducing time off work and/or childcare [7]. The study enabled the development of a maternity-specific framework of the domains of quality that appear most relevant to stakeholders in remote antenatal care: efficiency and timeliness; effectiveness; safety; accessibility; equity and inclusion; person-centredness and choice and continuity [37].

## Future directions addressing the evidence gaps

### Developing clinical pathways for SMBP as a system

The studies above have shown there remains a need for further evidence around antihypertensive treatment in pregnancy, including guidance on up-titration and optimal antihypertension medication in the context of SMBP, and the clinical significance of white coat (or masked) hypertension.

Successful implementation will require consideration of the shifts in workload between staff and pregnant women that SMBP will entail, as well as support for healthcare professionals to overcome barriers, such as long-held beliefs about accuracy of automated BP monitors in pregnancy and concerns about the foetal impact of tight BP control, despite recent evidence supporting this strategy [30, 31]. Trusted educational resources for both healthcare teams and pregnant people will be needed to support any such implementation. SMBP appears to work best with good relationships and communication between women and their health care professionals and this should be supported as an integral part of the intervention [22].

### Self-monitoring for multiple indicators

The new development of hypertension and pre-eclampsia are a substantial concern through pregnancy, and antenatal care includes monitoring for a range of indicators of evolving disease. The UDIP study described above showed that self-testing for proteinuria could support remote care of women with hypertension, and indeed around half of maternity units surveyed during the pandemic were also offering proteinuria self-testing in selected groups [28]. Companies are beginning to develop kits specially design for home testing in pregnancy and some studies in pregnancy are including additional interventions such as home foetal monitoring through cardiotocography and symptom checklists [38, 39]. Robust evaluation of the clinical outcomes of such multi-faceted interventions is awaited.

### Ensuring equity in research and implementation

The long-standing inequalities in maternity outcomes have been amplified and thrown into even sharper focus by the pandemic [40, 41]. Addressing the challenges of these inequalities requires a high-quality and inclusive evidence base [42]. Without this approach, there are risks that the introduction of new modes of antenatal care might compound the problems of marginalisation, disadvantage and clinical risk for women in some most at-risk groups [37, 42]. This evidence is currently lacking. While studies examining the use of remote monitoring and telehealth show promising results, as outlined above, they are not conclusive. Many have assessed only individual components of maternity care, are generally small scale and not reflective of diverse populations [43–46]. More generally, research focussed on digital health and access to care has remained very limited [47]. While we may well be witnessing a steady shift towards increasing inclusion of self-monitoring and remote care in antenatal care pathways, we need to focus on the potentially important implications that this shift has for access to care, and potentially on clinical outcomes [33].

## Summary and conclusions

These large SMBP trials, alongside shifts in care pathways introduced during the COVID-19 pandemic, have served to test the implementation of SMBP, but a further organisational shift is needed to see definitive benefit. While pregnant women are willing and able to use and interpret SMBP readings, current care pathways need to evolve further to facilitate potential impact on clinical outcomes. System changes can often be hard, and implementation of this complex intervention is not straightforward. This body of research has shown that, both outside of pregnancy and now for the pregnant population, the intervention needs to be multi-faceted and system-wide, beyond handing out BP monitors, in order to change clinical outcomes [4, 10, 11].

What is needed now is a system response that addresses evidence gaps such as the addressing home-clinic BP difference, understanding the most effective antihypertensive mediation and titration schedules to use in pregnancy while adequately considering issues of equity in research and implementation.

### Supplementary information

Supplemental data
