# Supplementary material for: Using self-monitoring to detect and manage raised blood pressure and pre-eclampsia during pregnancy: the BUMP research programme and its impact
Source: Hypertens Res. 2023 Dec 7;47(3):714–20. doi: 10.1038/s41440-023-01474-w (PMC10912026; doi:10.1038/s41440-023-01474-w)
Supplement: Supplementary file 1 — Supplemental data [file 41440_2023_1474_MOESM1_ESM.docx]

**Supplemental data**

(Using self-monitoring to detect and manage raised blood pressure and pre-eclampsia during pregnancy: The BUMP research programme and its impact)

**Supplemental Information: Guidance for Self-monitoring blood pressure during pregnancy**

All those who self-monitored their BP as part of the BUMP trials were provided with a monitor validated for use in pregnancy and pre-eclampsia, along with clear instructions for its use. They were asked to sit quietly and comfortably for one minute, then take two readings one minute apart, using the second reading to interpret the colour-coded charts provided. (Supplementary Figures 1 and 2) The charts provided instructions about how to act, and if the second reading was outside the expected range, this led to a request for a third reading (taken after 5 minutes), which, if still raised or low, would recommend that women contact their local maternity unit (giving a time frame for doing so). BP thresholds were equivalent to clinic thresholds (140/90 mmHg) based on pilot work and a systematic review. (1, 2)

Those with a higher-risk pregnancy (BUMP1) were asked to monitor three times a week. Those with hypertension (BUMP2) were asked to monitor their BP daily throughout their pregnancy.

In the trials, all those self-monitoring were given access to a telemonitoring system that would, upon entering a BP reading, automatically provide guidance based on the appropriate colour chart. The App also provided weekly messages designed to provide support and education throughout the trial. Clinical teams could access the BP readings and would receive alerts if women did not enter readings for serval days or had inputted high readings.

**Supplementary Figure 1:** Blood pressure interpretation chart for BUMP1 (higher-risk pregnancy)

| **LEVEL** | **BLOOD PRESSURE**  **/mmHg** | **ACTION** |
| --- | --- | --- |
| **HIGH** | SYS 150 or more  OR  DIA 100 or more | Your blood pressure is high  Sit quietly for 5 minutes then measure it again and send in the reading.  Contact your maternity unit for urgent assessment today (within 4 hours) and continue to monitor your BP daily. |
| **RAISED** | SYS 140-149  OR  DIA 90-99 | Your blood pressure is raised  Sit quietly for 5 minutes then measure it again and send in the reading.  If your repeated reading is raised please contact your maternity unit within 24 hours and continue to monitor your BP daily. |
| **HIGH NORMAL** | SYS 135-139  OR  DIA 85-89 | Your blood pressure is normal but moving towards the raised threshold  Sit quietly for 5 minutes then measure it again and send in the reading.  If your repeat reading is still high-normal, please monitor your blood pressure daily. |
| **NORMAL** | SYS 110-134  OR  DIA 70-84 | Your blood pressure is normal.  Continue blood pressure monitoring and your current care |
| **LOW** | SYS 109 or less  AND  DIA 69 or less | Your blood pressure is low. Repeat once more in 5 minutes.  If you are taking blood pressure medication, contact your maternity unit within 24 hours or within 4 hours if you feel unwell (e.g. dizzy or faint).  If you are not taking medication and you are feeling well this blood pressure does not need any further action. |

*First published in Dougal *et al*., 2020 (3) and used to develop RCOG guidelines for self-monitoring of BP during pregnancy.(4)

**Supplementary Figure 2:** Blood pressure interpretation chart for BUMP2 (Hypertensive pregnancy)

| **LEVEL** | **BLOOD PRESSURE**  **/mmHg** | **ACTION** |
| --- | --- | --- |
| **HIGH** | SYS 150 or more  OR  DIA 100 or more | Your blood pressure is high  Sit quietly for 5 minutes then measure it again and send in the reading.  Contact your maternity unit for urgent assessment today (within 4 hours) and continue to monitor your BP daily. |
| **RAISED** | SYS 140-149  OR  DIA 90-99 | Your blood pressure is raised  Sit quietly for 5 minutes then measure it again and send in the reading.  If your repeated reading is raised please contact your maternity unit within 24 hours and continue to monitor your BP daily. |
| **NORMAL** | SYS 110-139  OR  DIA 70-89 | Your blood pressure is normal.  Continue blood pressure monitoring and your current care |
| **LOW** | SYS 109 or less  AND  DIA 69 or less | Your blood pressure is low. Repeat once more in 5 minutes.  If you are taking blood pressure medication, contact your maternity unit within 24 hours or within 4 hours if you feel unwell (e.g. dizzy or faint).  If you are not taking medication and you are feeling well this blood pressure does not need any further action. |

*First published in Dougal *et al*., 2020 (3) and used to develop RCOG guidelines for self-monitoring of BP during pregnancy.(4)

1. Tucker KL, Bankhead C, Hodgkinson J, Roberts N, Stevens R, Heneghan C, et al. How Do Home and Clinic Blood Pressure Readings Compare in Pregnancy? Hypertension. 2018;72(3):686-94.

2. Tucker KL, Taylor KS, Crawford C, Hodgkinson JA, Bankhead C, Carver T, et al. Blood pressure self-monitoring in pregnancy: examining feasibility in a prospective cohort study. BMC Pregnancy Childbirth. 2017;17(1):442.

3. Dougall G, Franssen M, Tucker KL, Yu LM, Hinton L, Rivero-Arias O, et al. Blood pressure monitoring in high-risk pregnancy to improve the detection and monitoring of hypertension (the BUMP 1 and 2 trials): protocol for two linked randomised controlled trials. BMJ Open. 2020;10(1):e034593.

4. RCOG. Royal College of Obstetricians and Gynaecologists guidlines - Self-monitoring of blood pressure in pregnancy <https://www.rcog.org.uk/globalassets/documents/guidelines/2020-03-30-self-monitoring-of-blood-pressure-in-pregnancy.pdf2020>
